# Supplementary material for: Tuning structural isomers of phenylenediammonium to afford efficient and stable perovskite solar cells and modules
Source: Nat Commun. 2021 Nov 4;12:6394. doi: 10.1038/s41467-021-26754-2 (PMC8568940; doi:10.1038/s41467-021-26754-2)
Supplement: Supplementary file 1 — Supplementary Information [file 41467_2021_26754_MOESM1_ESM.pdf]

Tuning Structural Isomers of Phenylenediammonium to Afford Efficient and Stable Perovskite Solar Cells and Modules

Cheng Liu[1,2]†, Yi Yang[1,2]†, Kasparas Rakstys[3]*, Arup Mahata[4,5], Marius Franckevicius[6], Edoardo Mosconi[5], Raminta Skackauskaite[3], Bin Ding[2], Keith G. Brooks[2], Onovbaramwen Jennifer Usiobo[7], Jean-Nicolas Audinot[7], Hiroyuki Kanda[2], Simonas Driukas[6], Gabriele Kavaliauskaite[6], Vidmantas Gulbinas[6], Marc Dessimoz[2], Vytautas Getautis[3], Filippo De Angelis[4,8,9], Yong Ding[1,2]*, Songyuan Dai[1]*, Paul J. Dyson[2]* & Mohammad Khaja Nazeeruddin[2,10]*

†These authors contributed equally: Cheng Liu & Yi Yang

Supplementary Methods

Chemicals required for the synthesis were purchased from Sigma-Aldrich and TCI Europe and used as received without additional purification. ^{1}H NMR spectra were recorded at 400 MHz on a Bruker Avance III spectrometer with a 5 mm double resonance broad band BBO z-gradient room temperature probe, ^{13}C NMR spectra were collected using the same instrument at 101 MHz. The chemical shifts, expressed in ppm, were relative to tetramethylsilane (TMS). All the NMR experiments were performed at 25 °C. Reactions were monitored by thin-layer chromatography on ALUGRAM SIL G/UV254 plates and developed with UV light. Silica gel (grade 9385, 230–400 mesh, 60 Å, Aldrich) was used for column chromatography. Elemental analysis was performed with an Exeter Analytical CE-440 elemental analyser, Model 440 C/H/N/. MS were recorded on Waters SQ Detector 2 Spectrometer using electrospray ionization (ESI) technique. Lead iodide (PbI_2, 99.99%), lead bromide ($PbBr_2$, 98%), and phenethylammonium iodide (PEAI, >98.0%) were purchased from Tokyo Chemical Industry Co., LTD. Methylammonium iodide (MAI, >99.5%), cesium iodide (CsI, >99.9%), methylammonium chloride (MACl, >99.5%), formamidinium iodide (FAI, >99.5%) and Spiro-MeOTAD (>99.0%) were purchased from the Xi'an Polymer Light Technology Corp. All reagents were used as received without further purification. Synthetic route

for $PDEAI_2$ cations is shown in Supplementary Figure 1.

2,2'-(1,4-phenylene)diacetonitrile (1)

NaCN (1.85 g, 37.77 mmol) and 1,4-bis(bromomethyl)benzene (5.00 g, 18.94 mmol) were dissolved in EtOH (10 mL) and distilled H_2O (5 mL) and refluxed for 7 hours. After cooling to RT reaction mixture was extracted with EtOAc (3 x 100 mL), dried over anhydrous Na_2SO_4, filtered and solvent evaporated *in vacuo*. The product was purified by column chromatography on silica gel using 20-40% acetone in hexane. Yield 2.41 g (81%). ^1H NMR (400 MHz, Chloroform-*d*) δ 7.36 (s, 4H), 3.77 (s, 4H). ^{13}C NMR (101 MHz, $CDCl_3$) δ 130.48, 129.23, 117.95, 23.79. Anal. calcd for $C_{10}H_8N_2$: C 76.90; H 5.16; N 17.94; found: C 76.95; H 5.32; N 17.77. $C_{10}H_8N_2[M^+]$ exact mass = 156.07, MS (ESI) = 156.18.

2,2'-(1,3-phenylene)diacetonitrile (2)

NaCN (2.04 g, 41.62 mmol) and 1,3-bis(bromomethyl)benzene (5.50 g, 20.83 mmol) were dissolved in EtOH (10 mL) and distilled H_2O (5 mL) and stirred for 1 hour under reflux. After that reaction mixture was extracted with EtOAc (3 x 100 mL), dried over Na_2SO_4, filtered and solvent evaporated. The product was purified by column chromatography on silica gel using 20% acetone in hexane. Yield 2.20 g (67%). ^1H NMR (400 MHz, Chloroform-*d*) δ 7.45 – 7.37 (m, 1H), 7.34 – 7.28 (m, 3H), 3.77 (s, 4H). ^{13}C NMR (101 MHz, $CDCl_3$) δ 131.42, 130.36, 128.06, 127.82, 117.72, 23.81. Anal. calcd for $C_{10}H_8N_2$: C 76.90; H 5.16; N 17.96; found: C 75.78; H 5.37; N 17.65. $C_{10}H_8N_2[M^+]$ exact mass = 156.07, MS (ESI) = 154.96.

2,2'-(1,2-phenylene)diacetonitrile (3)

NaCN (1.85 g, 37.77 mmol) and 1,2-bis(bromomethyl)benzene (5.00 g, 18.94 mmol) were dissolved in EtOH (10 mL) and distilled H_2O (5 mL). After being refluxed for 3 hours, reaction mixture was

extracted with EtOAc and dried over Na_2SO_4 then distilled. The product was purified by column chromatography on silica gel using 10% acetone in hexane. Yield 1.70 g (57%). ^{1}H NMR (400 MHz, Chloroform-*d*) δ 7.50 – 7.37 (m, 4H), 3.78 (s, 4H). ^{13}C NMR (101 MHz, $CDCl_3$) δ 130.23, 129.79, 128.57, 116.84, 21.95. Anal. calcd for $C_{10}H_8N_2$: C 76.90; H 5.16; N 17.94; found: C 77.65; H 5.33; N 16.93. $C_{10}H_8N_2[M^+]$ exact mass = 156.07, MS (ESI) = 154.99.

1,4-phenylenediethylamine (4)

1 (1.00 g, 6.40 mmol) was dissolved in THF (50 mL) and a solution of 1 M borane tetrahydrofuran complex (45 mL, 542.69 mmol) was added dropwise under argon atmosphere and refluxed for 24 hours. The solution was cooled down in an ice bath and THF:H_2O (v:v; 1:1) (50 mL) was added dropwise leading to a white precipitate. The mixture was distilled to remove THF and then EtOH (100 mL) and concentrated H_2SO_4 (1 mL) were added. The mixture was refluxed for 30 min. The solvents were removed *in vacuo*, 1 M NaOH (100 mL) solution was added to neutralize the acid. The mixture was extracted with $CHCl_3$ (3 x 50 mL), dried over Na_2SO_4, filtered, and evaporated, leading to a white solid. The crude was used for the next step without further purification. Yield 1.05 g (99%). $C_{10}H_{16}N_2[M^+]$ exact mass = 164.13, MS (ESI) = 164.50.

1,3-phenylenediethylamine (5)

To a solution of **2** (1.10 g, 7.04 mmol) in THF (50 mL), 1 M borane tetrahydrofuran complex solution (45 mL, 542.69 mmol) was added dropwise under argon atmosphere and refluxed for 24 hours. The reaction mixture was cooled down to RT and placed in an ice bath. THF:H_2O (v:v; 1:1) (50 mL) was added dropwise to neutralize the complex, leading to a white precipitate. The mixture was distilled to remove THF and EtOH (100 mL) and concentrated H_2SO_4 (1 mL) were added. The mixture was refluxed for 30 min. The solvents were removed *in vacuo*, 1 M NaOH (100 mL)

solution was added to neutralize the acid. The mixture was extracted with $CHCl_3$, dried over Na_2SO_4, filtered, and distilled, leading to a white solid. The crude was used for the next step without further purification. Yield 1.15 g (99%). $C_{10}H_{16}N_2[M^+]$ exact mass = 164.13, MS (ESI) = 164.47.

1,2-phenylenediethylamine (6)

To a solution of **3** (0.85 g, 5.44 mmol) in THF (50 mL), 1 M borane solution in THF (45 mL) was added dropwise under argon atmosphere and the mixture was refluxed for 24 hours. The solution was cooled down in an ice bath and THF:H_2O (v:v; 1:1) (50 mL) was added leading to a white precipitate. The mixture was distilled to remove THF and then EtOH (100 mL) and concentrated H_2SO_4 (1 mL) were added. The mixture was refluxed for 30 min. The solvents were removed *in vacuo*, 1 M NaOH (100 mL) solution was added to neutralize the acid. The mixture was extracted with $CHCl_3$, dried over Na_2SO_4, filtered, and distilled, leading to a white solid. The crude was used for the next step without further purification. Yield 0.62 g (69%). $C_{10}H_{16}N_2[M^+]$ exact mass = 164.13, MS (ESI) = 164.24.

1,4-(phenylene)di(ethylammonium) iodide (*p*-PDEAI₂)

4 (1.20 g, 7.30 mmol) was dissolved in CH_3OH (60 mL) and reaction flask was placed in an ice bath. HI (57 %, 12 mL) was added dropwise under argon atmosphere. The flask was covered with foil to avoid light exposure and stirred overnight at RT. Reaction mixture was evaporated *in vacuo* and the obtained crude was dissolved in methanol and precipitated into 20-fold excess of Et_2O. The product was filtered, washed with Et_2O and dried to yield 2.39 g of yellowish solid (77%). ^1H NMR (400 MHz, DMSO-d_6) δ 7.74 (s, 6H), 7.23 (s, 4H), 3.08 – 3.00 (t, J = 9.6 Hz, 4H), 2.85 – 2.81 (t, J = 9.9, 6.3 Hz, 4H). ^{13}C NMR (101 MHz, DMSO) δ 135.00, 128.26, 38.23, 31.98. Anal. calcd for $C_{10}H_{18}I_2N_2$: C 28.59; H 4.32; N 6.67; I 60.42; found: C 30.02; H 4.24; N 6.4. $C_{10}H_{18}I_2N_2[M^+]$ exact

mass = 419.96. MS (ESI) = 418.82.

1,3-(phenylene)di(ethylammonium) iodide (*m*-PDEAI_2)

5 (1.00 g, 6.08 mmol) was dissolved in CH_3OH (50 mL) under argon atmosphere. HI (57 %, 10 mL) was added dropwise while stirring the mixture in an ice bath. The flask was covered with foil to avoid light exposure and left to stir for 24 hours. Then the reaction mixture was evaporated *in vacuo* and the obtained crude was dissolved in methanol and precipitated into 20-fold excess of Et_2O. Precipitate was filtered, washed with Et_2O and dried to obtain the product as yellowish solid. Yield 0.62 g (23%). ^{1}H NMR (400 MHz, DMSO-d_6) δ 7.75 (s, 6H), 7.32 (t, J = 7.4 Hz, 1H), 7.16 (d, J = 8.3 Hz, 3H), 3.06 (dd, J = 9.5, 6.4 Hz, 4H), 2.85 (dd, J = 9.7, 6.3 Hz, 4H). ^{13}C NMR (101 MHz, DMSO) δ 137.53, 129.06, 128.96, 127.13, 38.89, 32.94. Anal. calcd for $C_{10}H_{18}I_2N_2$: C 28.59; H 4.32; N 6.67; I 60.42; found: C 28.9; H 4.34; N 6.6. $C_{10}H_{18}I_2N_2[M^+]$ exact mass = 419.96. MS (ESI) = 418.83.

1,2-(phenylene)di(ethylammonium) iodide (*o*-PDEAI_2)

6 (0.50 g, 3.04 mmol) was dissolved in CH_3OH (25 mL) and HI (57 %, 5 mL) was added dropwise under argon atmosphere while stirring the mixture in an ice bath. The flask was covered with foil to avoid light exposure and left to stir for 24 hours. Reaction mixture was evaporated *in vacuo* and the obtained crude dissolved in methanol and precipitated into 20-fold excess of Et_2O. Precipitate was filtered, washed with Et_2O and dried to obtain the product as yellowish solid. Yield 1.00 g (78%). ^{1}H NMR (400 MHz, DMSO-d_6) δ 7.78 (s, 6H), 7.26 (s, 4H), 3.08 – 2.97 (m, 4H), 2.97 – 2.84 (m, 4H). ^{13}C NMR (101 MHz, DMSO) δ 135.50, 129.61, 129.40, 127.27, 45.39, 40.20, 38.89, 29.69. Anal. calcd for $C_{10}H_{18}I_2N_2$: C 28.59; H 4.32; N 6.67; I 60.42; found: C 30,25; H 4,26; N 6,2. $C_{10}H_{18}I_2N_2[M^+]$ exact mass = 419.96. MS (ESI) = 418.83.

Supplementary Computational Details

First-principles calculations based on density functional theory (DFT) are carried out as implemented in the PWSCF Quantum-Espresso package[1]. Geometry optimization, including dispersion correction[2], was performed using GGA-PBE[3] level of theory and the electrons-ions interactions were described by ultrasoft pseudo-potentials with electrons from I 5s, 5p; N, C 2s, 2p; H 1s; Pb, 6s, 6p, 5d; shells explicitly included in calculations. Plane wave basis set cutoffs for the smooth part of the wave functions and the augmented density were 50 and 400 Ry, respectively. Both ions and volume were relaxed during the optimization of the bulk 2D perovskites. Formation energies ($E_{formation}$) of the bulk 2D perovskites were calculated using the following formula: $E_{formation} = E_{total} - E_{Pb2+} - E_{I-} - E_{cationic\text{-}molecule}$, where E_{total}, E_{Pb2+}, E_{I-} and $E_{cationic\text{-}molecule}$ are the total energy of the bulk 2D perovskite, Pb^{2+} cation, isolated I^- anion and isolated cationic molecule, respectively with the corresponding numbers of ions involves for the bulk 2D perovskites of four formula units. E_{Pb2+} was calculated from the energy of the bulk PbI_2 in its P_{3m1} crystal phase. Cationic molecules were optimized in 30×30×30 Å^3 isolated box with Gamma kpoint sampling.

Perovskite surface was modeled by cutting 2 × 2 slabs from the bulk tetragonal $MAPbI_3$ crystal structure, which exposes MAI-terminated (001) surface. The 2 × 2 slabs (a = b = 17.7 Å) were optimized with Gamma point sampling. The experimental $MAPbI_3$ cell parameters were employed to build a periodic supercell in the x and y directions, whereas a vacuum of more than 10 Å is introduced along the surface truncation direction. The reaction thermodynamics of the cation replacement process were calculated by considering the following equilibrium: MAI-terminated slab + cationic molecule = cation inserted MAI-terminated slab + 2 MA molecules. Adsorption energy (E_{ads}) is calculated using the following formula: $E_{ads} = E_{slab+cationic\ molecule\ adsorbed} - E_{slab} - E_{cationic}$

molecule, where $E_{\text{slab+cationic molecule adsorbed}}$ represents the total energy of the adsorbed cationic molecules with the slab, E_{slab} is the total energy of the bare slab, and $E_{\text{cationic molecule}}$ is the energy of the isolated cationic molecule in a large supercell. The absolute values of the adsorption energies are large because of the adsorption of a gas-phase cationic molecule.

Supplementary Figures

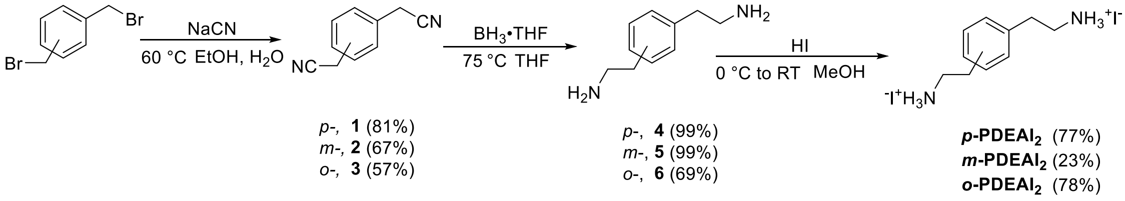

Supplementary Figure 1: Synthetic route for PDEAI₂ cations.

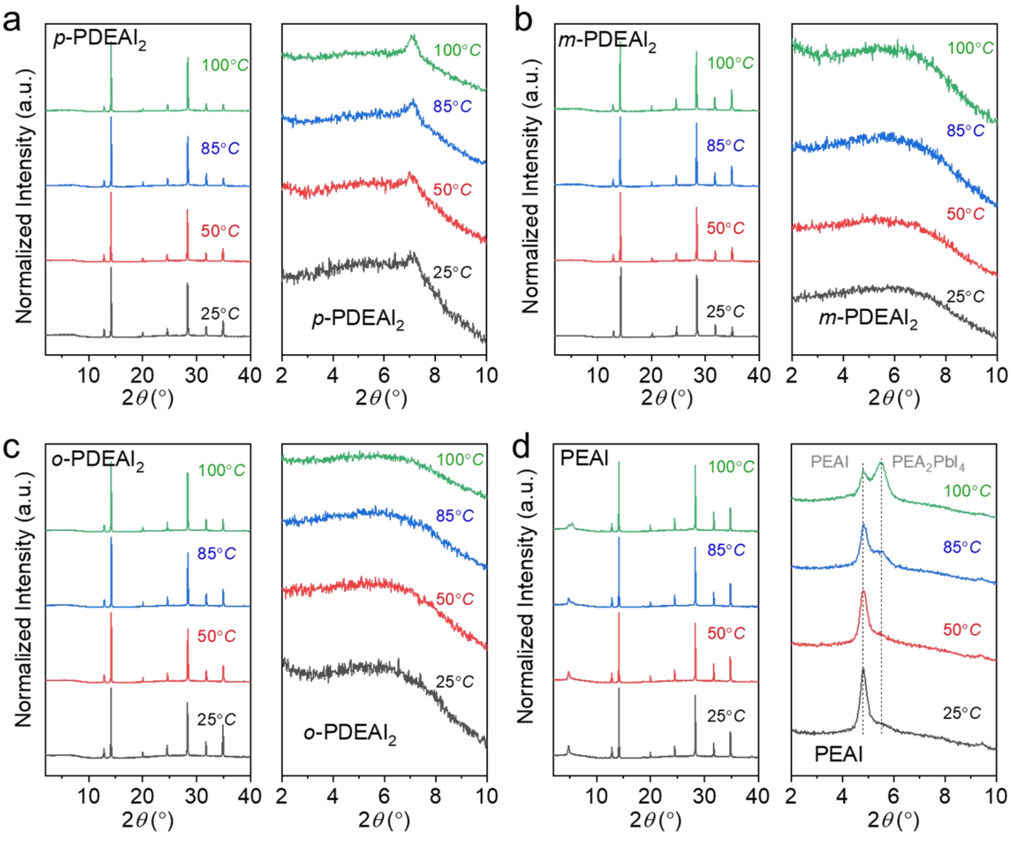

Supplementary Figure 2. XRD patterns for perovskite films with organic halide salt passivation at different

annealing temperatures. **a** *p*-PDEAI$_2$ passivation. **b** *m*-PDEAI$_2$ passivation. **c** *o*-PDEAI$_2$ passivation. **d** PEAI passivation.

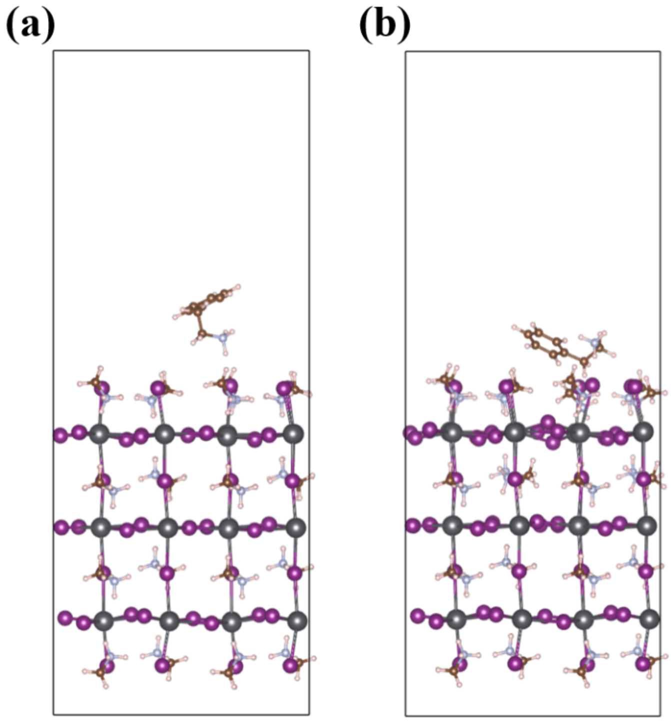

Supplementary Figure 3. Optimized structures for the adsorption of PEA cation on the perovskite surface. Adsorption through **a** -NH_3 group and **b** through phenyl group.

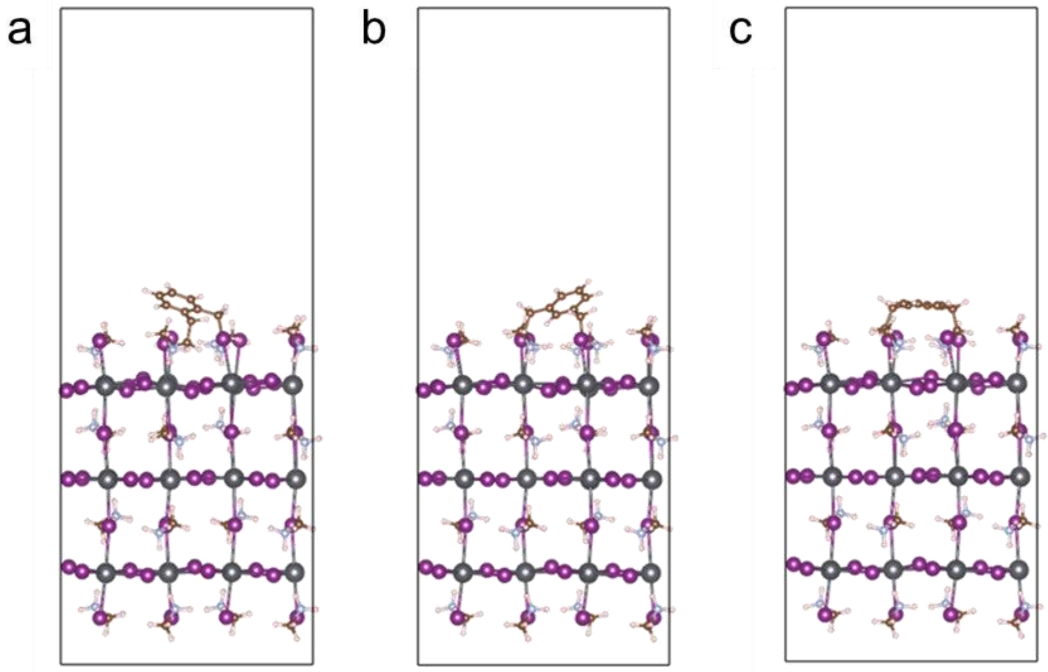

Supplementary Figure 4. The formation possibility of 2D perovskite for higher layer thickness. Optimized

structures for the insertion of **a** *o*-PDEAI$_2$, **b** *m*-PDEAI$_2$ and **c** *p*-PDEAI$_2$ cations by replacing two methylammonium cations of perovskite surface.

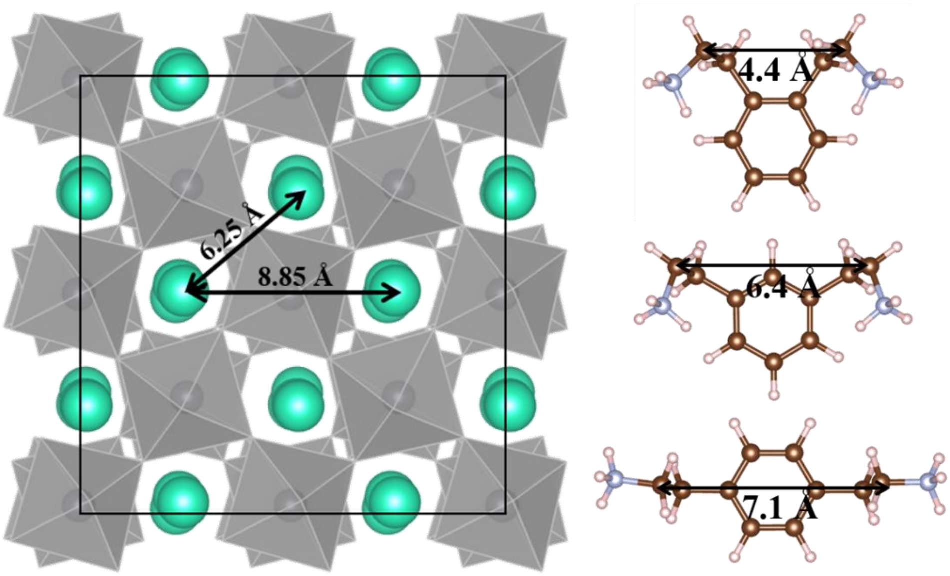

Supplementary Figure 5. Mismatching distance of PDEAI$_2$ and perovskite surface. Distance between the octahedra of MAI-terminated MAPbI$_3$ surface and the distance between the -CH$_2$-CH$_2$-NH$_3^+$ groups of the 2D cations. The cyan color ball is placed on the middle of the C-N bond, thus fairly represents the distance between the octahedra.

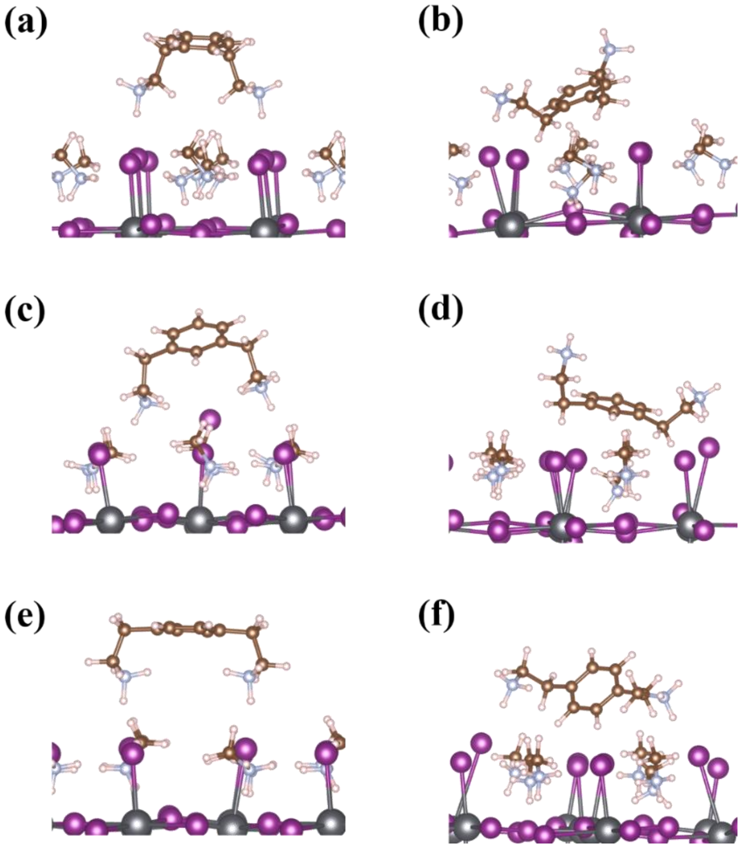

Supplementary Figure 6. Zoomed structures. Optimized structures for the adsorption of **a-b** ortho-, **c-d** meta- and

e-f para cation on the perovskite surface. Left and right panel represents the adsorption through ammonium and phenyl group, respectively.

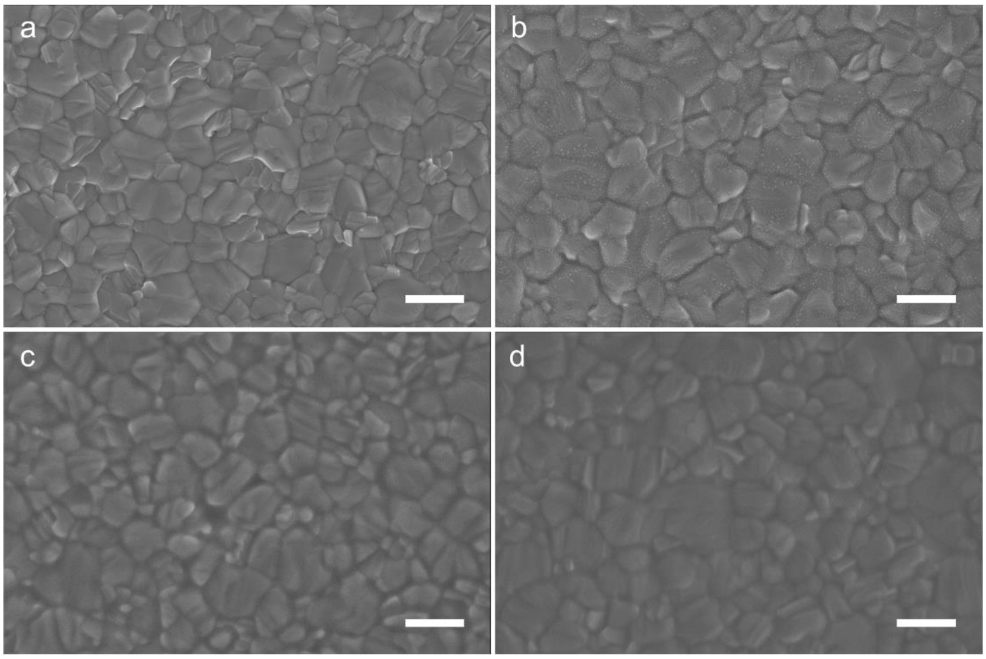

Supplementary Figure 7. Morphology of perovskite films after PDEAI₂ deposition. SEM images of the **a** pure perovskite surface, **b** with *p*-$PDEAI_2$, **c** with *m*-$PDEAI_2$ and **d** with *o*-$PDEAI_2$ passivation. Scale bars, 1 μm.

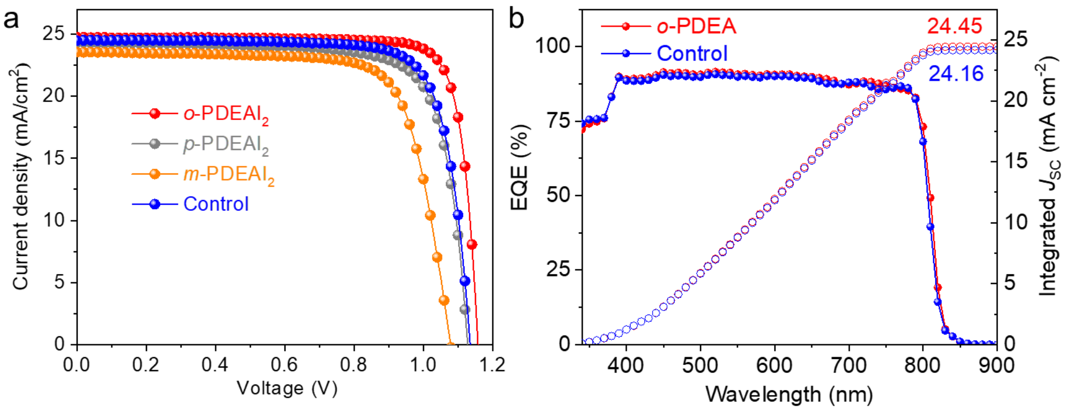

Supplementary Figure 8. Photovoltaic performance for devices with $PDEAI_2$ passivation. a *J-V* characteristics of the devices with different isomer passivation. **b** EQE spectra of the best-performing control and *o*-$PDEAI_2$-passivted devices.

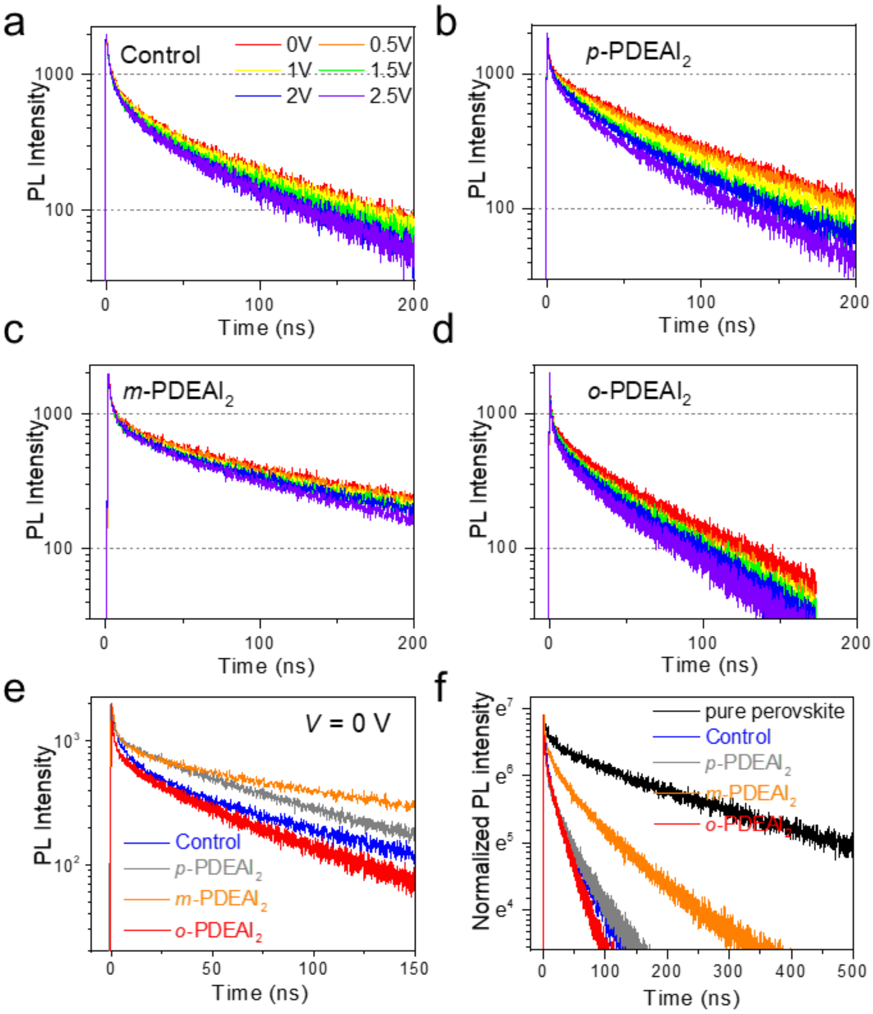

Supplementary Figure 9. Voltage-dependent transient PL and transient photocurrent investigations. a-e PL decay kinetics at different applied voltages of the control and $PDEAI_2$ passivated devices. **f** Transient PL kinetics of perovskite layers on glass substrates.

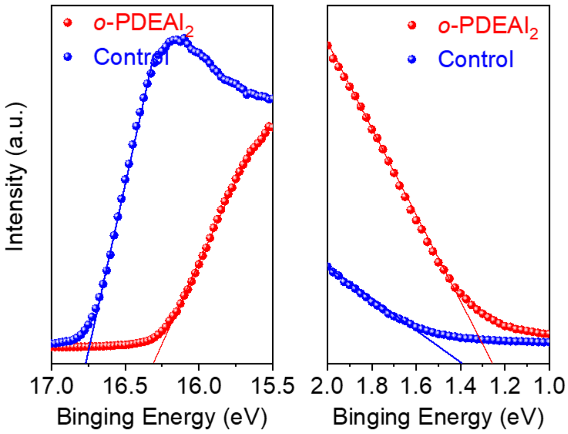

Supplementary Figure 10. Surface band structure. UPS results of the pure and *o*-$PDEAI_2$-passivated perovskite surface.

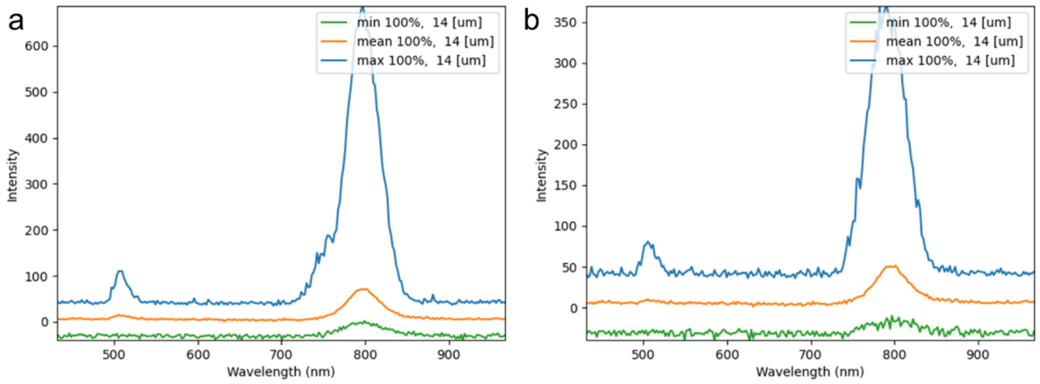

Supplementary Figure 11. Film uniformity revealed by the CL characterization. CL spectra of **a** control and **b** *o*-PDEAI$_2$-passivated perovskite film.

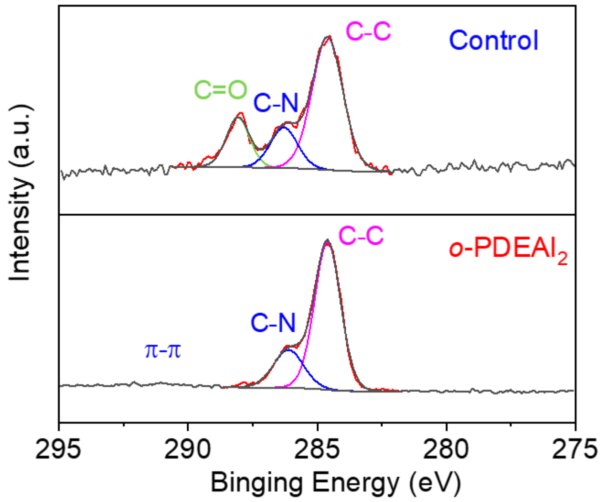

Supplementary Figure 12. The presence of *o*-PDEAI$_2$ on the perovskite surface. High-resolution XPS spectra of C 1*s* for the control and *o*-PDEAI$_2$-passivated perovskite film.

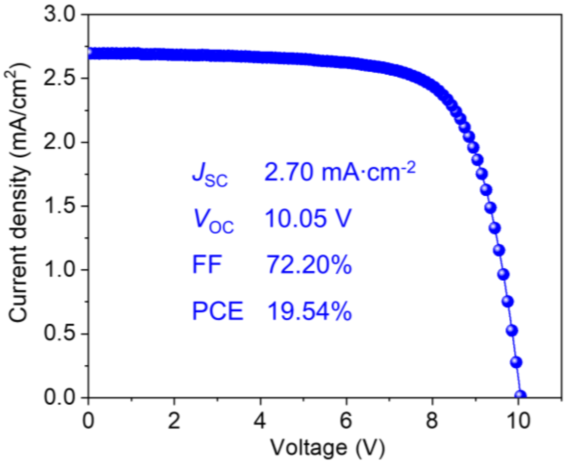

Supplementary Figure 13. Photovoltaic performance of perovskite solar modules. *J*-*V* characteristics of the control module with an active area of 26.00 cm^2.

Supplementary Tables

Supplementary Table 1. Formation energies of the 2D and quasi-2D perovskites.

Structures	Energy (eV)	
	2D perovskite	quasi-2D perovskite
p-$PDEAI_2$	-7.19	-0.76
m-$PDEAI_2$	-7.22	-0.97
o-$PDEAI_2$	-6.41	-0.59

Supplementary Table 2. Adsorption energies of the PEA and PDEA cations on perovskite surface considering both ethyl ammonium and phenyl adsorption modes.

Structures	Energy (eV)	
	$-CH_2CH_2NH_3^+$	phenyl
PEA	-9.28	-9.90
p-PDEA	-9.76	-10.04
m-PDEA	-10.50	-9.51
o-PDEA	-10.00	-9.53

Supplementary Table 3. Summary of the device performance for the control and $PDEAI_2$-passivated (2 mg mL^{-1}) devices.

Condition	V_{OC} (V)	J_{SC} (mA cm^{-2})	FF	PCE (%)
Control	1.112±0.01	24.24±0.25	0.769±0.02	20.71±0.66
Best	1.135	24.49	0.790	21.94
p-$PDEAI_2$	1.097±0.03	24.20±0.32	0.765±0.02	20.30±0.60
Best	1.127	24.34	0.770	21.09
m-$PDEAI_2$	0.993±0.05	23.80±0.57	0.701±0.05	16.74±1.57
Best	1.079	23.55	0.750	19.63
o-$PDEAI_2$	1.141±0.01	24.52±0.31	0.802±0.01	22.45±0.64
Best	1.157	24.75	0.835	23.92

Supplementary Table 4. Summary of the device performance with different $PDEAI_2$ concentrations.

Condition	Concentration (mg mL^{-1})	V_{OC} (V)	J_{SC} (mA cm^{-2})	FF	PCE (%)
Control	0	1.135	24.49	0.790	21.94
p-$PDEAI_2$	1	1.129	24.31	0.766	21.05
	2	1.127	24.34	0.770	21.09
	3	1.131	23.90	0.767	20.70
	4	1.117	23.87	0.765	20.43
	5	1.115	23.83	0.751	19.94

	1	1.098	23.77	0.762	19.87
	2	1.079	23.55	0.750	19.63
m-$PDEAI_2$	3	1.063	24.14	0.720	18.48
	4	1.061	23.95	0.703	17.91
	5	1.048	22.04	0.708	16.32
	1	1.147	24.64	0.822	23.23
	2	1.157	24.75	0.835	23.92
o-$PDEAI_2$	3	1.153	24.60	0.832	23.63
	4	1.151	24.46	0.816	22.98
	5	1.148	24.37	0.799	22.36

Supplementary Table 5. Summary of the fitting parameters of the perovskite films on glass substrates.

Condition	A_1 [%]	τ_1 [ns]	A_2 [%]	τ_2 [ns]
Pure perovskite w/o HTL	32.5	14.3	67.5	284.4
Control with HTL	65.6	4.5	34.4	47.2
p-$PDEAI_2$ with HTL	57.1	2.5	42.9	38.2
m-$PDEAI_2$ with HTL	45.6	6.6	54.4	90.0
o-$PDEAI_2$ with HTL	38.3	2.4	61.7	28.9

Supplementary Table 6. Overview of the reported perovskite solar modules so far.

Year	Ref.	Active Area (cm^2)	Designated Area (cm^2)	GFF (%)	Number of Subcells	J_{SC} ($mA\cdot cm^2$)	V_{OC} (V)	FF (%)	PCE (%)
2018	4	–	33	93.4	17	1.15	18.19	72.1	15.3
2019	5	8	–	–	4	5.82	3.98	68	15.79
2020	6	–	36.1	85	10	1.77	11.19	64.91	12.85
2020	7	–	36	–	10	1.80	10.4	67.67	12.67
2020	8	7.92	–	85.7	4	5.66	4.55	76.2	19.6
2020	9	–	25.49	90.8	7	3.03	7.52	78.59	17.88
2020	10	2	–	90	6	3.43	6.35	71	15.5
2020	11	–	22.4	91	7	2.99	7.64	72.9	16.6
2021	12	8	–	–	4	5.63	4.17	71.11	16.69
2021	13	36.6	–	90.3	7	3.04	7.44	71	16.06
2021	14	42.8	–	–	14	1.50	16.05	70.89	17.05
2021	15	–	35.8	–	10	2.04	11.70	77.3	18.5
2021	16	17.1	–	–	6	3.80	6.88	78.1	20.42
2021	17	27.14	–	92	8	3.08	8.71	75.41	20.2
2021	This work	26	–	90.2	9	2.71	10.30	76.4	21.36

Supplementary References

1 Giannozzi, P. et al. QUANTUM ESPRESSO: a modular and open-source software project

for quantum simulations of materials. *J. Phys. Condens. Matter* **21**, 395502 (2009).

2 Grimme, S., Antony, J., Ehrlich, S. & Krieg, H. A consistent and accurate ab initio parametrization of density functional dispersion correction (DFT-D) for the 94 elements H-Pu. *J. Chem. Phys.* **132**, 154104 (2010).

3 Perdew, J. P., Burke, K. & Ernzerhof, M. Generalized gradient approximation made simple. *Phys. Rev. Lett.* **77**, 3865 (1996).

4 Deng, Y. *et al.* Surfactant-controlled ink drying enables high-speed deposition of perovskite films for efficient photovoltaic modules. *Nature Energy* **3**, 560-566 (2018).

5 Xu, Z. *et al.* Br-containing alkyl ammonium salt-enabled scalable fabrication of high-quality perovskite films for efficient and stable perovskite modules. *Journal of Materials Chemistry A* **7**, 26849-26857 (2019).

6 Ru, P. *et al.* High electron affinity enables fast hole extraction for efficient flexible inverted perovskite solar cells. *Advanced Energy Materials* **10**, 1903487 (2020).

7 Li, E. *et al.* Synergistic Coassembly of Highly Wettable and Uniform Hole-Extraction Monolayers for Scaling-up Perovskite Solar Cells. *Advanced Functional Materials* **30**, 1909509 (2020).

8 Du, M. *et al.* High-Pressure Nitrogen-Extraction and Effective Passivation to Attain Highest Large-Area Perovskite Solar Module Efficiency. *Advanced Materials* **32**, 2004979 (2020).

9 Ren, A. *et al.* Efficient perovskite solar modules with minimized nonradiative recombination and local carrier transport losses. *Joule* **4**, 1263-1277 (2020).

10 Rolston, N. *et al.* Rapid open-air fabrication of perovskite solar modules. *Joule* **4**, 2675-2692 (2020).

11 Liu, Z. *et al.* A holistic approach to interface stabilization for efficient perovskite solar modules with over 2,000-hour operational stability. *Nature Energy* **5**, 596-604 (2020).

12 Chen, R. *et al.* Crown Ether-Assisted Growth and Scaling Up of FACsPbI3 Films for Efficient and Stable Perovskite Solar Modules. *Advanced Functional Materials* **31**, 2008760 (2021).

13 Huang, H.-H. *et al.* A simple one-step method with wide processing window for high-quality perovskite mini-module fabrication. *Joule* **5**, 958-974 (2021).

14 Nia, N. Y. *et al.* Beyond 17% stable perovskite solar module via polaron arrangement of tuned polymeric hole transport layer. *Nano Energy* **82**, 105685 (2021).

15 Chen, S., Xiao, X., Gu, H. & Huang, J. Iodine reduction for reproducible and high-performance perovskite solar cells and modules. *Science Advances* **7**, eabe8130 (2021).

16 Bu, T. *et al.* Lead halide–templated crystallization of methylamine-free perovskite for efficient photovoltaic modules. *Science* **372**, 1327-1332 (2021).

17 Deng, Y. et al. Defect compensation in formamidinium–caesium perovskites for highly efficient solar mini-modules with improved photostability. *Nature Energy* **6**, 633-641, (2021).
