# Supplementary material for: Tuning structural isomers of phenylenediammonium to afford efficient and stable perovskite solar cells and modules
Source: Nat Commun. 2021 Nov 4;12:6394. doi: 10.1038/s41467-021-26754-2 (PMC8568940; doi:10.1038/s41467-021-26754-2)
Supplement: Supplementary file 3 — Solar Cells Reporting Summary [file 41467_2021_26754_MOESM3_ESM.pdf]

nature research

Corresponding author(s): Mohammad Khaja Nazeeruddin

Solar Cells Reporting Summary

Nature Research wishes to improve the reproducibility of the work that we publish. This form is intended for publication with all accepted papers reporting the characterization of photovoltaic devices and provides structure for consistency and transparency in reporting. Some list items might not apply to an individual manuscript, but all fields must be completed for clarity.

For further information on Nature Research policies, including our data availability policy, see Authors & Referees.

Experimental design

Please check: are the following details reported in the manuscript?

1. Dimensions

Item	Yes	No	Details
Area of the tested solar cells	☒	☐	Characterization part in Method.
Method used to determine the device area	☒	☐	Characterization part in Method.

2. Current-voltage characterization

Item	Yes	No	Details
Current density-voltage (J-V) plots in both forward and backward direction	☒	☐	See Figure 3b.
Voltage scan conditions<br>*For instance: scan direction, speed, dwell times*	☒	☐	Characterization part in Method.
Test environment<br>*For instance: characterization temperature, in air or in glove box*	☒	☐	Characterization part in Method.
Protocol for preconditioning of the device before its characterization	☒	☐	Characterization part in Method.
Stability of the J-V characteristic<br>*Verified with time evolution of the maximum power point or with the photocurrent at maximum power point; see ref. 7 for details.*	☒	☐	Fig. 3b and Fig. 5g

3. Hysteresis or any other unusual behaviour

Item	Yes	No	Details
Description of the unusual behaviour observed during the characterization	☒	☐	"Passivation effects on photovoltaic performance" section in the Main text
Related experimental data	☒	☐	See Fig. 3b.

4. Efficiency

Item	Yes	No	Details
External quantum efficiency (EQE) or incident photons to current efficiency (IPCE)	☒	☐	See Supplementary Figure 7b.
A comparison between the integrated response under the standard reference spectrum and the response measure under the simulator	☒	☐	"Passivation effects on photovoltaic performance" section in the Main text
For tandem solar cells, the bias illumination and bias voltage used for each subcell	☐	☒	No tandem solar cell was reported.

5. Calibration

Item	Yes	No	Details
Light source and reference cell or sensor used for the characterization	☒	☐	Characterization part in Method.
Confirmation that the reference cell was calibrated and certified	☒	☐	Characterization part in Method.

Calculation of spectral mismatch between the reference cell and the devices under test

- [ ] Yes
- [x] No

No spectral mismatch calculation was performed.

6. Mask/aperture

Size of the mask/aperture used during testing

- [x] Yes
- [ ] No

Characterization part in Method.

Variation of the measured short-circuit current density with the mask/aperture area

- [ ] Yes
- [x] No

We didn't measure the solar cells with apertures of different sizes.

7. Performance certification

Identity of the independent certification laboratory that confirmed the photovoltaic performance

- [ ] Yes
- [x] No

No certification.

A copy of any certificate(s)
Provide in Supplementary Information

- [ ] Yes
- [x] No

No certification.

8. Statistics

Number of solar cells tested

- [x] Yes
- [ ] No

Caption for Fig. 3c.

Statistical analysis of the device performance

- [x] Yes
- [ ] No

See Fig. 3c.

9. Long-term stability analysis

Type of analysis, bias conditions and environmental conditions
For instance: illumination type, temperature, atmosphere humidity, encapsulation method, preconditioning temperature

- [x] Yes
- [ ] No

Fig. 5 d-g and Characterization part in Method.
